# The Influence of Change-Related Organizational and Job Resources on Employee Change Engagement

**DOI:** 10.3389/fpsyg.2022.910206

**Published:** 2022-06-13

**Authors:** Simon L. Albrecht, Sean Connaughton, Michael P. Leiter

**Affiliations:** ^1^School of Psychology, Faculty of Health, Deakin University, Melbourne, VIC, Australia; ^2^Melbourne Business School, Melbourne, VIC, Australia

**Keywords:** change engagement, change engagement model, change-related organizational resources, change-related job resources, employee attitudes to change

## Abstract

Employee attitudes to change are key predictors of organizational change success. In this article, change engagement is defined as the extent to which employees are enthusiastic about change, and willing to actively involve themselves in ongoing organizational change. A model is tested showing how change-related organizational resources (e.g., senior leader support for change and organizational change climate) influence change engagement, in part through their influence on change-related job resources. Confirmatory Factor Analysis (CFA) and Structural Equations Modeling (SEM) results yielded good fit to the data in two independent samples: 225 Australian working professionals, and 201 employees from a Prolific sample. As proposed, change-related organizational resources (modeled as a higher order construct) were positively associated with higher order change-related job resources. Change-related job resources were positively associated with change engagement. In contrast to expectations, organizational resources were not directly associated with change engagement. Instead, change-related job resources fully mediated the relationship. Overall, the study provides empirical support for new measures of organizational change resources and employee change engagement. By drawing from well-established models in the change and engagement literatures, the study provides a promising research direction for those interested in further understanding positive employee attitudes to organizational change. Practical implications and future research opportunities are discussed.

## Introduction

Organizational change is recognized as a constant within the contemporary world of work (Tsaousis and Vakola, 2018; Karasvirta and Teerikangas, 2022). The successful navigation of ongoing organizational change is recognized as key to competitive advantage and organizational survival (Burnes, 2004; By, 2005; Fugate et al., 2012). Organizations that are open and receptive to continuous change attract, select, and retain employees who are more energized by and open to change than employees in organizations that have a focus on stability and intermittent change (Weick, 2000; Thundiyil et al., 2015; Beus et al., 2020).

Despite organizational change being “the new normal,” it is commonly claimed that a large proportion of change initiatives fail (Burnes, 2011; Choi, 2011; Pasmore, 2011). Failure rates of 20–40% to 70–80% have been reported (Beer and Nohria, 2000; Weiner et al., 2008; Burke, 2011; Burnes, 2011; Choi, 2011). The variation in failure rates has been partially attributed to imprecision in the measurement of success criteria, and differing stakeholder perceptions about what constitutes successful change (see Hughes, 2011). It is important that success criteria are informed by relevant theories and research evidence so that organizations can optimally understand the factors that promote and maximize the likelihood of successful organizational change (Oreg et al., 2011; Straatmann et al., 2016).

**FIGURE 1:**
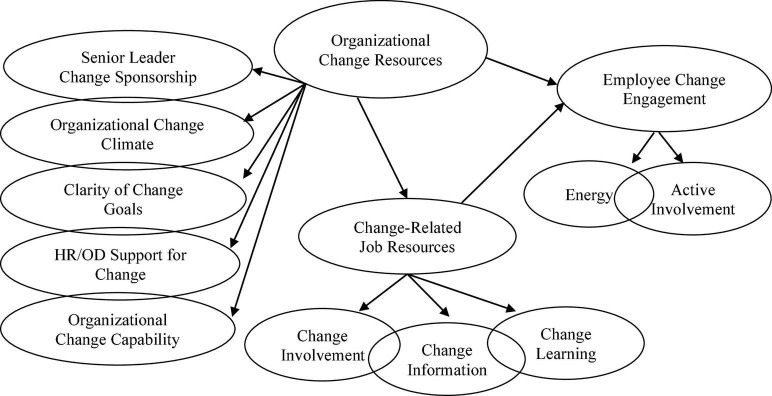
Proposed model. Items, errors, and implied indirect effects not shown for ease of representation.

In this article, we aim to make three contributions to the change management literature. Firstly, we aim to establish “change engagement” as a construct and as a measure. Secondly, we identify five organizational change-related resources that we propose will directly influence change engagement. Thirdly, and as shown in Figure 1, we establish whether change-related organizational resources influence change engagement through their influence on three change-related job resources that have previously been shown influence employee attitudes to change.

### Employee Attitudes to Change

Numerous researchers have argued that employee attitudes to change are fundamental to the success or failure of organizational change (Bartunek et al., 2006; Rafferty et al., 2013). Research has shown that successful organizational change depends on employees feeling positive about, and willing to adopt, any proposed changes (Feldman, 2000; Wanous et al., 2000; Oreg, 2006; Van Emmerik et al., 2009). Mathews and Linski (2016) argued that it is particularly important to focus on positive attitudes because “the negative and deficiency-based approach used to frame the subject of employee resistance to change seems counterproductive to the end goal of learning how to positively address resistance and implement change successfully” (p. 963). Nevertheless, negative employee attitudes to change have been linked to adverse change outcomes (Schweiger and Denisi, 1991; Wanberg and Banas, 2000; Thundiyil et al., 2015). Overall, there is strong research evidence to suggest it is important to identify what influences employee attitudes to change in order to facilitate and optimize employee receptiveness to change, and therefore the likely success of organizational change. It is important that employees are open to change irrespective of whether they are front-line employees, team leaders, supervisors, or managers (Devos et al., 2007).

### Resources and Employee Attitudes to Organizational Change

Job-Demands Resources theory (JD-R; Bakker and Demerouti, 2014, 2017), engagement theory (Kahn, 1990), and Conservation of Resources theory (COR; Hobfoll et al., 2018) predict that the number and nature of resources available to employees will have an important influence on their attitudes, motivation, and behavior. JD-R theory, for instance, predicts that a range of job and personal resources will lead to employee engagement and additional positive employee and organizational outcomes. In support of the theory, meta-analytic studies (e.g., Crawford et al., 2010; Halbesleben, 2010) have shown that job resources such as job autonomy, job involvement and learning and development opportunities lead to employee engagement, job satisfaction, organizational citizenship behavior, and task performance.

Beyond the influence of job resources, there is increasing evidence to show that organizational resources also influence employee motivation, attitudes, and performance (Biggs et al., 2014; Barrick et al., 2015; Albrecht et al., 2018). Organizational resources have been defined as system-level aspects of the organizational environment, that are not role specific, and that directly or indirectly influence employee attitudes, well-being, and performance (Albrecht et al., 2018). Albrecht et al. (2020) showed that a range of organizational resources (e.g., senior leadership engagement, human resources (HR) management practices, and clarity of organizational goals) had a direct influence on job resources and employee engagement. Similarly, using longitudinal data, Biggs et al. (2014) showed that the organizational resource “strategic alignment” had a positive influence on employee engagement over a 12-month time lag. Research has also shown that job resources partially mediate the influence of organizational resources on motivational, well-being and performance outcomes (Biggs et al., 2014; Barrick et al., 2015; Albrecht et al., 2020). Albrecht et al. (2020), for example, showed that clarity of organizational goals, strategic alignment, organizational autonomy, organizational adaptivity, human resource practices, and senior leadership behavior indirectly influenced engagement through job resources (e.g., job autonomy, job variety, supervisor support, and development opportunities).

The current study is focused on the influence of change-related organizational and job resources on employee attitudes to change. By adopting a change resources perspective, and by extending more generic motivational theories such as the JD-R to the context of organizational change, we suggest that employee perceptions of change-related organizational and job resources will predict positive attitudes to change. Our focus is on work-related resources, and we do not address the potential influence of employee disposition to change, change-related personal resources, or change-related job demands. Below, we introduce, describe, and define the constructs of change engagement, job resources, and organizational resources. Consistent with tenets of COR theory, we propose that change-related organizational resources, job resources, and change engagement can be modeled as higher order constructs. COR recognizes that “resources do not exist individually but travel in packs, or caravans, for both individuals and organizations” (Hobfoll et al., 2018, p. 107), and that multiple resources are needed to achieve positive motivational and performance outcomes.

### Change Engagement

Albrecht (2021) argued that change engagement provides a more energized and motivational expression of positive change-related attitudes than constructs such as openness to change, readiness for change, commitment to change, and willingness to engage in organizational change. Change engagement can be defined as “an enduring and positive work-related psychological state characterized by a genuine enthusiasm and willingness to support, adopt and promote organizational change” (Albrecht et al., 2020, p. 4). This definition applies the two fundamental qualities of employee engagement – positive energy and active involvement (González-Romá et al., 2006; Macey and Schneider, 2008) – to the context of organizational change. As such, and drawing from well-established theoretical models of job-related affect (Russell, 2003; Oreg et al., 2018), both engagement and change engagement can be characterized as high-arousal and high-valence constructs. Constructs characterized by activated positive affect imply a predisposition to action (Frijda, 1986) and a willingness to invest energy and involvement in organizational change.

As modeled in Figure 1, change engagement is proposed to consist of two sub-dimensions: change energy and active involvement. “Energy” captures the activated positive affect and employee enthusiasm required for the successful implementation and adoption of organizational change. “Active Involvement” captures a more behaviorally oriented willingness to strive toward the achievement of successful organizational change. Although constructs such as affective reactions to change and willingness to change (Bouckenooghe et al., 2009) have previously been proposed and measured, these constructs have not been brought together with psychosocial antecedents within a well-validated JD-R framework (Bakker and Demerouti, 2017).

### Change-Related Job Resources

Change-related job resources have been defined as the psychological, physical, technological, informational, and social supports and supplies available to employees to help them successfully adapt to, and adopt, organizational change (Albrecht et al., 2020). Information about change, involvement in change, and opportunities for learning and development are change-related job resources that have consistently been associated with positive employee attitudes to change (e.g., Coch and French, 1948; Wanberg and Banas, 2000; Van Emmerik et al., 2009; Straatmann et al., 2016). Wanberg and Banas (2000) used longitudinal data to show that positive perceptions about change information and a higher degree of participation in change were related to positive employee perceptions about organizational change. Van Emmerik et al. (2009), using the JD-R model, reported that opportunities for professional development were positively associated with favorable evaluations of organizational change.

Although numerous researchers have focused on the influence of change-related job resources, less research has been focused on how “upstream” organizational resources (Dollard and Bakker, 2010) such as senior leadership sponsorship of change, organizational change climate, and HR practices influence employee perceptions of change-related resources and their attitudes to change. Additionally, very few change researchers have proposed a model that includes employee attitudes to change, change-related job resources, and change-related organizational resources within a coherent theoretical framework (e.g., JD-R). As per Figure 1, the primary purpose of the current study is to identify if and how change-related organizational resources influence positive employee attitudes to change.

### Change-Related Organizational Resources

Key organizational resources that have been linked to attitudes to change include organizational climate, senior leader sponsorship and support for change, clarity of change goals, HR support for change, and overall organizational change capability. Each of these change-related organizational resources is briefly outlined below.

#### Organizational Change Climate

Organizational climate is an important determinant of successful and sustained organizational change (Schneider et al., 1996, p. 18). Organizations where employees share perceptions that change is inevitable, necessary, and legitimate have been shown to succeed at organizational change (e.g., Denison et al., 2013; Costanza et al., 2016). This is because employees within flexible and adaptive organizational cultures and climates develop a mindset that can cope with changing job responsibilities, organizational strategies, and structures (Eby et al., 2000; Rafferty and Simons, 2005). When employees share positive perceptions towards change, the resulting organizational climate will make it more likely that individual employees, irrespective of their level or role, will report being open to and enthusiastic about the legitimacy and inevitability of organizational change (Sagiv and Schwartz, 2007).

#### Senior Leadership Sponsorship of Change

Senior leadership’s active support is widely recognized as fundamental to the success of organizational change (e.g., Kotter, 1990; Armenakis and Bedeian, 1999; Beer, 2021). Senior leaders need to promote, communicate, and reinforce the need for ongoing change (Bordia et al., 2004). Resources that senior leaders provide to support successful organizational change can include a clear change purpose and vision, clear communication of information about change, and active sponsorship and support for change (Rafferty et al., 2013; Ten Have et al., 2017; Ford et al., 2021). Yukl et al. (2019) argued that change leaders need to envision change, advocate for change, and encourage innovation. Despite calls for more collaborative and shared models of change leadership (e.g., Pearce and Sims, 2002; By, 2021), the relationship between senior leader support for change and employee attitudes to change is a “truth” that has been established across many empirical studies (Burke, 2014).

#### Clarity of Organizational Change Goals

Clarity of organizational goals as an organizational resource has been shown to influence employee engagement (Albrecht et al., 2018). Clarity of organizational change goals can include information about expected benefits associated with change, the criteria for successful change, and information about how proposed change goals relate to wider organizational goals (Milliken, 1987; Wanberg and Banas, 2000; Elving, 2005). Organizational change goals that are communicated from the organizational level (e.g., CEO communications) can be more effective at promoting positive employee attitudes to change than when communicated by an immediate supervisor (Allen et al., 2007; Devos et al., 2007). Overall, research shows that clear and timely communication about the overarching goals of organizational change helps reduce employee uncertainty and enhances employee openness to change (Eby et al., 2000; Allen et al., 2007). Additionally, and drawing from engagement theory (Bakker and Xanthopoulou, 2013; Albrecht et al., 2015), clarity of organizational change goals will likely be positively associated with change engagement both directly and indirectly through change-related job resources.

#### Human Resources Support for Change

Human resources change supports are the administrative, policy, procedural and structural resources that organizations make available to employees to structure and support their employment conditions, wellbeing, and performance. HR practices have been shown to have a direct influence on employee attitudes to change (Whelan-Berry and Somerville, 2010). More specifically, training, recruitment, performance appraisal, and reward and recognition systems that align with organizational change goals and capability (e.g., training in how to cope with change) result in positive employee attitudes to change (Armenakis et al., 1999; Whelan-Berry and Somerville, 2010; Fugate, 2012). Just as HR practices influence an organization’s engagement climate (Barrick et al., 2015), change focused HR practices and initiatives will influence employee perceptions about change-related job resources and employee engagement in change. HR change supports will equally impact front-line, team leader, supervisor, and manager openness and engagement in change.

#### Organizational Change Capability

To be able to adapt to continually changing environmental contexts, “successful organizations [need to] develop the internal capacity to implement change faster and more effectively than their competitors” (Miller, 2004, p. 9). Organizational change capability, as an organizational resource, reflects “a combination of managerial and organizational capabilities that allows an enterprise to adapt more quickly and effectively than its competition to changing situations” (Judge and Douglas, 2009, pp. 635–636). Similar to constructs such as organizational change readiness (Weiner, 2009; Shea et al., 2014), change capability requires organizations to have policies and procedures in place that prime them to be “change ready,” frameworks and methodologies that support a common understanding of how to manage change, and the physical, technological, and HR it needs to deal effectively with ongoing change. Although a limited number of theorists and researchers have described and defined change capability (e.g., Beer, 1999; Eisenhardt and Martin, 2000; Meyer and Stensaker, 2006), the influence of organizational change capability on employee attitudes to change has not been extensively researched.

In summary, although there is accumulating research evidence suggesting that organizational resources are important in shaping employee attitudes to change, the evidence is neither well-established nor theoretically integrated. Drawing from engagement theory, COR theory, and organizational change literature (e.g., Barrick et al., 2015; Albrecht et al., 2018), the current research aims to extend the limited amount of change research that has focused on organizational-level change resources, and to address calls for more theory to be applied to examination of organizational change (By, 2005). Based on the preceding review, and consistent with Figure 1, it is proposed that change related organizational resources, modeled as a higher order construct, will have a significant and positive association with employee change engagement and change-related job resources. Furthermore, it is proposed that the association between organizational change resources and employee change engagement will be partially mediated by change-related job resources. The proposed partial mediation parallels results reported by Albrecht et al. (2018) when examining the indirect effects of organizational resources on employee engagement. The modeling is also consistent with arguments that resources at multiple levels of analysis inter-relate to influence individual motivational outcomes (Goh et al., 2022) and are consistent with Albrecht et al.’s (2015) integrated model showing how organizational resources such as organizational climate directly influence the employee experience of job resources and engagement.

## Method

### Participants and Procedure

Data were drawn from two samples. For Sample 1, contacts within the researchers’ primary professional networks were email-invited to participate in an on-line survey and to refer the invitation to other eligible participants within their networks. Eligible participants needed to be 18 years or older and working full- or part-time for a minimum of 3 months in an organization of 15 or more employees that had experienced organizational change within the past year. The email invitation informed participants about the purpose of the study, assured them of their confidentiality, and that the procedure was granted ethics approval by the first author’s University ethics committee.

Of the 225 participants from Sample 1, 57.4% were female. Ages ranged from 22 to 63 years with a mean of 39 years. Tenure ranged from 6 months to 40 years, with a mean of 5.9 years. Respondents worked in organizations of varying size (15–10,000^+^ employees), as team members (41.3%), senior managers (18.8%), team leaders (8.1%), and executives (4.9%). Most participants (83.9%) reported experiencing a “moderate amount” to a “great deal” of organizational change during the past 12 months.

The participants for Sample 2 were sourced using Prolific.^1^ Prolific enables researchers to access paid participants that meet specific inclusion criteria. Recent research has shown that data derived from Prolific “has similar psychometric properties and produces criterion validities that generally fall within the credibility intervals of existing meta-analytic results from conventionally sourced data” (Walter et al., 2019, p. 425). Beyond having a Prolific record of providing quality survey responses, participants also needed to meet the same eligibility criteria as Sample 1.

Of the 201 Prolific participants, 50.7% were female. Ages ranged from 18 to 66 years, with a mean of 36 years. Tenure ranged from 6 months to 40 years, with a mean of 5.9 years. Respondents worked in organizations of varying size (10–100,000 employees) as team members (54.2%), senior managers (5.0%), managers (16.4%), team leaders (17.9%), and executives (1.5%). Most participants (78.7%) reported experiencing a moderate to a great deal of organizational change during the past 12 months. Prolific participants were informed before commencing the survey that they needed to attend carefully to, and complete, all survey questions in order to receive the agreed terms of payment.

### Measures

The items indicating the 10 first-order constructs shown in Figure 1 were anchored on a seven-point Likert scale (1 = strongly disagree, 7 = strongly agree). Table 1 shows the items used in the analyses and their Confirmatory Factor Analysis (CFA) standardized loadings for Sample 1 and Sample 2. The scale means, standard deviations, reliabilities, and correlations are shown in Table 2.

**TABLE 1:** Respecified CFA items and their loadings Sample 1 and Sample 2.

Scale	Item	Loadings Sample 1	Loadings Sample 2
***Change-related organizational resources***			
CCLIM1	People understand there are legitimate reasons for needing to change.	0.751	0.643
CCLIM2	People are positive about change.	0.761	0.688
CCLIM3	People realize that change is inevitable.	0.813	0.799
CCLIM4	People recognize that change is necessary in order to continually improve.	0.806	0.958
SLS2	Senior leaders consistently communicate that ongoing organizational change is important to the success of our organization.	0.874	0.855
SLS3	Senior leaders actively encourage employees to embrace organizational change.	0.898	0.819
SLS4	Senior leaders are seen to be active sponsors and supporters of change.	0.848	0.844
COCG1	As we continue to go through ongoing change, the future direction of the organization is clearly communicated to everyone.	0.905	0.871
COCG2	People have a good understanding of what organization changes are trying to achieve.	0.885	0.878
COCG3	As we experience ongoing organizational change, people still have a strong sense of where the organization is going.	0.942	0.919
COCG4	Everyone who works here is well aware of how organizational changes fit in with the long-term plans and direction of the organization.	0.817	0.920
HRSC1	The HR department (or equivalent) provides people with sufficient opportunities for training and development to successfully go through organizational changes.	0.807	0.844
HRSC2	As we continue to go through change, HR (or equivalent) keeps people informed about how the changes are going.	0.890	0.868
HRSC3	HR (or equivalent) helps ensure the right people with the right skills are in the best positions to support ongoing change.	0.846	0.860
HRSC4	HR (or equivalent) uses processes that ensure everyone is treated fairly as we go through organizational changes.	0.782	0.822
OCC1	Our organization has the policies and procedures in place that enable us to be “change ready.”	0.862	0.907
OCC2	Our organization has frameworks and methodologies that help us manage change.	0.915	0.948
OCC3	Our organization has the resources we need to deal effectively with ongoing change.	0.903	0.846
***Change-related job resources***			
JRINV2	I am provided with opportunities to participate in the discussions that are had prior to the implementation of organizational changes.	0.869	0.882
JRINV3	I have opportunities to participate in the planning of organizational changes.	0.964	0.958
JRINV4	I have some input into how changes are implemented in this organization.	0.901	0.911
JRINFO1	I am clearly informed about the reasons underlying proposed organizational changes.	0.852	0.836
JRINFO2	I am informed about the implications of all proposed changes.	0.872	0.850
JRINFO3	I am regularly informed about how changes are progressing.	0.915	0.847
JRINFO4	The information I receive about change adequately answers questions I may have regarding change.	0.854	0.897
JRLO1	As we go through organizational change, I have opportunities to keep refreshing my competencies and capabilities.	0.902	0.890
JRLO2	Organizational change provides me with opportunities to develop new skills.	0.945	0.925
JRLO3	Organizational change offers me the possibility to learn new things.	0.937	0.928
***Change engagement – energy***			
CE-E_E1	I am enthusiastic about change in this organization.	0.854	0.893
CE-E_E2	I feel energized when we are going through change.	0.915	0.892
CE-E_E3	I feel positive about changes when they occur in this organization.	0.901	0.920
***Change engagement – active involvement***			
CE-I_I4	I strive as hard as I can to contribute positively to change initiatives in this organization.	0.828	0.842
CE-I_I5	I actively involve myself in changes that take place in this organization.	0.900	0.848
CE-I_I6	I strive to make sure change is implemented successfully in this organization.	0.874	0.926

*CCLIM, organizational change climate; SLCS, senior leader change sponsorship; COCG, clarity of organizational change goals; HRSC, human resources support for change; JRINV, job-resource involvement; JRINFO, job-resource information; JRLO, job-resource learning opportunities; CE-E, change engagement-energy; CE-I, change engagement-involvement.*

**TABLE 2:** Means, standard deviations (SD), Cronbach’s alpha (α), and correlations among first-order variables in the respecified measurement models Sample 1 (*N* = 225; below diagonal) and Sample 2 (*N* = 201; above diagonal).

	Sample 1			Sample 2												
Variable	Mean	SD	α	Mean	SD	α	1	2	3	4	5	6	7	8	9	10
1. Change engagement energy	4.88	1.44	0.92	4.45	1.46	0.93	–	0.72	0.33	0.22**	0.50	0.52	0.35	0.64	0.65	0.59
2. Change engagement striving	5.54	1.22	0.90	5.06	1.36	0.90	0.75	–	0.31	0.20**	0.43	0.40	0.33	0.51	0.51	0.61
3. Org change climate	5.20	1.21	0.86	5.16	1.11	0.85	0.32	0.36	–	0.49	0.49	0.51	0.53	0.48	0.33	0.39
4. Snr leader sponsorship	4.92	1.67	0.90	5.18	1.34	0.87	0.24	0.15*	0.56	–	0.62	0.45	0.59	0.51	0.29	0.24**
5. Clarity of org goals	4.58	1.64	0.94	4.68	1.53	0.94	0.41	0.32	0.54	0.74	–	0.67	0.65	0.79	0.68	0.47
6. HRM practices	4.25	1.64	0.90	4.34	1.54	0.91	0.42	0.30	0.31	0.58	0.65	–	0.62	0.74	0.60	0.54
7. Org change capability	4.30	1.70	0.92	4.88	1.40	0.93	0.32	0.25	0.46	0.64	0.65	0.66	–	0.53	0.34	0.37
8. Job change information	4.44	1.64	0.93	4.48	1.53	0.92	0.48	0.41	0.52	0.66	0.84	0.69	0.66	–	0.78	0.61
9. Job change involvement	4.16	1.96	0.93	3.82	1.85	0.94	0.49	0.46	0.30	0.34	0.42	0.50	0.39	0.63	–	0.61
10. Learning opportunities	5.21	1.49	0.95	4.94	1.56	0.94	0.46	0.47	0.41	0.39	0.41	0.49	0.42	0.53	0.50	–

All correlations significant at p ≤ 0.001 except * < 0.05, ** < 0.01.

#### Organizational Change Resources

Organizational change climate was measured with four items self-developed or adapted from Armenakis et al. (2007) and Paré et al. (2011). Senior leader change sponsorship was measured with five items adapted from Albrecht et al. (2018), Paré et al. (2011), and Judge and Douglas (2009). HR change support was measured with four items adapted from Gould-Williams and Davies (2005). Clarity of organizational change goals was measured with five items adapted from Albrecht et al. (2018) and Patterson et al. (2005). Organizational change capability was measured with three items self-developed or adapted from Miller (2004).

#### Job-Level Change Resources

Change-related learning opportunities were measured with three items adapted from Paré et al. (2011) and Armenakis et al. (2007). Change involvement was measured with five items adapted from Wanberg and Banas (2000). Change information was measured with four items adapted from Bouckenooghe et al. (2009), Miller et al. (1994), and Wanberg and Banas (2000).

#### Employee Change Engagement

The change engagement items were drawn from Albrecht et al. (2020) and adapted from existing engagement measures (e.g., González-Romá et al., 2006; Rich et al., 2010) and change measures (e.g., Bouckenooghe et al., 2009; Tsaousis and Vakola, 2018). The enthusiasm and active involvement sub-scales were each measured with three items (see Table 1).

### Data Analytic Approach

The data analytic strategy was based in Anderson and Gerbing’s (1988) widely used two-step approach. The approach involves first using CFA to assess the proposed measurement model against a range of established goodness-of-fit indices: Chi-square (χ^2^ not significant), Chi-square to degrees of freedom ratio (χ^2^/df < 2); TLI close to 0.95, CFI close to 0.95, SRMR close to 0.08, and RMSEA close to 0.06 (Hu and Bentler, 1999; Jackson et al., 2009). RMSEA values less than 0.08 and CFI values above 0.90 have also been suggested to represent acceptable fit (e.g., Browne and Cudeck, 1993; MacCallum et al., 1996; Byrne, 2001).

The data analysis strategy also involved assessing the influence of common method variance (CMV) (Podsakoff et al., 2012) and testing the invariance of the measurement model across both samples. Having established a defensible measurement model, the second step in the two-step approach involved using Structural Equations Modeling (SEM) to test the fit of the proposed model (see Figure 1).

## Results

### Measurement Model

Confirmatory Factor Analysis of the 10 factor first-order measurement model in Sample 1 yielded slightly less than acceptable fit (Model χ^2^ = 1,213.219, df = 657, χ^2^/df = 1.847, TLI = 0.924, CFI = 0.932, SRMR = 0.0615, RMSEA = 0.061, RMSEA 90% CI = 0.056–0.067). The TLI, CFI, and RMSEA, although close, did not meet strict fit criteria. Acknowledging that proposed measurement models rarely fit without subsequent modification (Anderson and Gerbing, 1988), modification indices were examined to identify items that contributed most to model misspecification. Given that a minimum of three to four items are sufficient to define a construct (Jöreskog and Sörbom, 1993), five lower loading items with relatively high modification indices were deleted (two from change involvement, two from senior leadership, and one from clarity of organizational change goals).

The respecified CFA yielded improved fit (Model χ^2^ = 828.927, df = 482, χ^2^/df = 1.720, TLI = 0.943, CFI = 0.951, SRMR = 0.0554, RMSEA = 0.057, RMSEA 90% CI = 0.050–0.063). All standardized loadings were significant (*p* < 0.001), ranging from 0.751 to 0.964 (see Table 1). Although the Chi-square ratio, CFI, and SRMR were at, or better than their criterion values, the TLI and RMSEA were not strictly at criteria. However, given that the modification indices did not suggest fixing or freeing parameters to improve model fit, and given that rule-of-thumb cut-off fit criteria need not be too strictly applied (Hu and Bentler, 1998; Heene et al., 2011), the respecified model provided acceptable fit to the data.

Testing for CMV using procedures recommended by Podsakoff et al. (2012) showed that standardized loadings for only 8 of the 34 items in the respecified model decreased by more than 0.25 when a common latent factor was included in the estimation of the model. Ten of the 34 item loadings decreased less than 0.10, and 29 items decreased less than 0.30. The three items with a decrease larger than 0.50 were from HR change support factor. Overall, the influence of method effects was shown to be modest given that all but one of the loadings remained statistically significant after the common latent factor was included in the model (Elangovan and Xie, 2000; Podsakoff et al., 2012).

The re-specified model also yielded generally acceptable fit in Sample 2: χ^2^ = 903.332, df = 482, χ^2^/df = 1.971, TLI = 0.924, CFI = 0.935, SRMR = 0.0526, RMSEA = 0.066, RMSEA 90% CI = 0.059–0.073). All standardized loadings were significant (*p* < 0.001), ranging from 0.643 to 0.958 (see Table 1). Despite not meeting strict fit criteria, the indices met accepted criteria (TLI > 0.90, CFI > 0.93, SRMR < 0.08, RMSEA < 0.08).

To assess the equivalence of the measures across both samples, cross-validation or invariance analyses were conducted. As a first step, the baseline test of the form of the model provided acceptable fit to the data (χ^2^ = 1,732.31, df = 964, χ^2^/df = 1.797, TLI = 0.934, CFI = 0.943, RMSEA = 0.043 CI 90%: 0.040–0.047). Next, even though Table 1 shows similar loadings, constraining the loadings to be equal across both samples resulted in a significant change in Chi-square relative to the baseline model (Δχ^2^ = 47.117, df = 24, *p* = 0.003). However, only three of the loadings were not statistically equivalent across the two samples, and freeing the equality constraints on the three non-invariant loadings (CCLIM3, SL3, and OCC3, see Table 1) resulted in a non-significant change in Chi-square (Δχ^2^ = 18.316, df = 21, *p* = 0.63). Therefore, given that invariance is indicated unless the majority of item loadings are non-invariant (Vandenberg and Lance, 2000), the results suggest partial invariance. Despite Table 2 showing very similar correlations across both samples, constraining the covariances to be equal, while maintaining the equality condition for all loadings, also resulted in a significant change in Chi-square (Δχ^2^ = 98.192, df = 55, *p* < 0.05). Seven of the 45 covariances were not invariant. Six of the seven non-invariant associations involved senior leadership sponsorship of change, and the one other non-invariant covariance involved change information and organizational change capability. Overall, and as per about a third of studies that test for measurement invariance (Putnick and Bornstein, 2016), the results suggest partial invariance of the measurement model.

**FIGURE 2:**
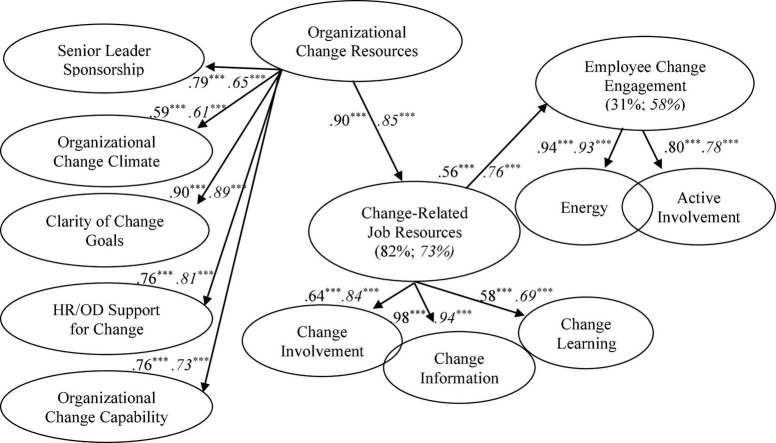
Respecified model; standardized parameter estimates; percent variance explained (Sample 2 in italics). Items, errors, and indirect effects not shown for ease of representation. ***p < 0.001.

Table 2 shows the means, standard deviations, Cronbach’s α, and correlations among the first-order variables included in the respecified CFA for Sample 1 and Sample 2. The table shows that all standardized loadings were greater than 0.63, all alpha reliabilities exceeded the recommended minimum criterion of 0.70 (Nunnally and Bernstein, 1994), and the magnitude of the bivariate correlations did not suggest multicollinearity. Consistent with the invariance results described above, the means, standard deviations, alphas, and correlations across both samples were very similar. Additionally, the standardized loadings of the first-order factors on organizational resources (0.59–0.90), job resources (0.64–0.98), and change engagement (0.80–0.94), provided support for the higher order modeling of the three constructs (see Figure 2).

Having established an acceptable measurement model with CFA, SEM was then used to test the fit of the proposed structural model (see Figure 1). The structural model using Sample 1 data yielded reasonably acceptable fit, albeit using less strict criteria: χ^2^ = 1,024.529, df = 547, χ^2^/df = 1.873, TLI = 0.927, CFI = 0.933, SRMR = 0.0785, RMSEA = 0.062, RMSEA 90% CI = 0.057–0.068. Organizational resources had a significant direct effect on job resources (β = 0.913, *p* < 0.001), and job resources had a significant direct effect on change engagement (β = 1.209, *p* < 0.001). However, organizational resources did not have a significant direct effect on change engagement (*p* = 0.069). Therefore, the path from organizational resources to change engagement was deleted and a re-specified SEM was estimated. The re-specified and more parsimonious model (see Figure 2) yielded improved fit: χ^2^ = 1,029.014, df = 548, χ^2^/df = 1.878, TLI = 0.927, CFI = 0.933, SRMR = 0.0741, RMSEA = 0.063, RMSEA 90% CI = 0.057–0.068. The parameters from organizational resources to job resources (β = 0.899, *p* < 0.001), and from job resources to change engagement (β = 0.559, *p* < 0.001) remained significant. Furthermore, even though no direct effect from organizational resources to change engagement was modeled, bootstrapping procedures showed that organizational resources had a significant indirect effect on change engagement through job resources (β = 0.503 [90% CI: 0.349–0.632], *p* = 0.009). Overall, the model explained 81% of the variance in job resources and 31% of the variance in change engagement. The high percentage variance explained in job resources by organizational resources no doubt contributed to the non-significant direct effect from organizational resources to change engagement in the proposed model.

For Sample 2, the Prolific sample, the structural model yielded broadly acceptable fit: Model χ^2^ = 1,026.641, df = 515, χ^2^/df = 1.993, TLI = 0.914, CFI = 0.921, SRMR = 0.0741, RMSEA = 0.070, RMSEA 90% CI = 0.064–0.077. Organizational resources had a significant direct effect on job resources (β = 0.853, *p* < 0.001), and job resources had a significant direct effect on change engagement (β = 0.761, *p* < 0.001). As with Sample 1, bootstrapping procedures showed that higher order organizational resources had a significant indirect effect on change engagement through job resources (β = 0.649 [90% CI: 0.505–0.763], *p* = 0.015). Overall, the model explained 73% of the variance in job resources and 58% of the variance in change engagement.

The final step in the data analysis process involved conducting relative weights analyses (RWA; Tonidandel et al., 2009; Tonidandel and LeBreton, 2015) to identify the percentage contribution of variance in change engagement explained by the first-order predictors. RWA analyses derive weights that decompose the overall model *R*^2^ across a set of predictors such that the sum of the weights equals the variance explained (Rafferty and Minbashian, 2019). RWA procedures have practical utility in that they identify the relative importance of predictors and, therefore, the “key drivers” of a construct.

Table 3 shows the relative importance in percentage terms for each of the organizational and job-level predictors. Overall, the RWA results showed that, in both samples, job level change resources accounted for most of the variance in change engagement, with change involvement and learning opportunities explaining the largest percent of the variance. Senior leader sponsorship of change, organizational change climate, and organizational change capability, despite significant bivariate correlations, accounted for non-significant or very little variance in either of the change engagement sub-dimensions.

**TABLE 3:** Relative weights analyses showing percent of variance explained in change engagement energy and change engagement active involvement in Sample 1 and Sample 2.

	Change engagement energy		Change engagement active involvement	
	Sample 1 (%)	Sample 2 (%)	Sample 1 (%)	Sample 2 (%)
Org change climate	8	5	15	6
Senior leader sponsorship	3	1	4	1
Clarity of org goals	10	9	10	8
HRM practices	13	10	5	7
Org change capability	4	5	3	6
Job change information	14	19	11	14
Job change involvement	27	27	17	17
Learning opportunities	22	24	26	40
Total variance explained	32	46	31	37

## Discussion

This study aimed to determine the extent to which a range of change-related organizational resources and change-related job resources were associated with employee change engagement. In contrast to previous studies that have included only a relatively narrow range of change-related organizational resources (e.g., Biggs et al., 2014), five resources were examined based on research highlighting their influence on positive attitudes to organizational change. The resources included organizational change climate, senior leadership sponsorship of change, HR change support, clarity of organizational change goals, and organizational change capability.

In partial support of the proposed modeling, and consistent with elaborated JD-R models and theory (Albrecht et al., 2018), change-related organizational resources were shown to be directly associated with change-related job resources. The considerable influence that organizational resources can have on employees’ change experiences was reflected in the sizable proportion of variance in job resources that was explained by organizational resources in both samples (81 and 73%).

In contrast to expectations, change-related organizational resources did not have a significant direct effect on employee change engagement. This may be partially attributable to the sizable direct influence of organizational resources on job resources, and the relatively strong influence of change-related job resources on change engagement. Additionally, researchers have argued that effects are likely to be stronger for constructs that are matched at their level of analysis (e.g., de Jonge and Dormann, 2006). Therefore, change-related organizational resources, such as senior leadership support, may be expected to have a stronger association with organizational outcomes such as organizational change commitment (Herscovitch and Meyer, 2002) as opposed to individual outcomes such as employee change engagement.

Despite the absence of a direct effect to change engagement, change-related organizational resources had a strong and significant indirect effect on employee change engagement through change-related job resources in both samples. As such, change-related organizational resources were found to have an important and fully mediated upstream influence on change engagement through change-related job resources. The results therefore suggest the need for a combination of organizational-level and job-level resources to optimally influence employee change engagement (Albrecht et al., 2020). As such, organizations can potentially realize positive outcomes by investing in system-level practices that are supportive of change, that help clarify organizational change goals, and that develop an organizational climate supportive of change. By doing so, it becomes more likely that employees will feel informed about change, involved in change, and learn and develop through change. As a result, employees will feel more enthusiastic about change and actively strive toward the achievement of successful organizational change; thereby increasing the likelihood of successful organizational change.

Consistent with the finding that the higher order change-related organizational resources did not directly influence employee change engagement, the RWA analyses demonstrated that a number of change-related organizational resources did not explain significant variance in change engagement when considered along with change-related job resources. Despite significant, albeit modest bivariate correlations, the RWA analyses showed that senior leader sponsorship and support of change, for example, did not explain significant variance in either sub-dimension of change engagement in either sample. This result contradicts much of the change management orthodoxy that defines senior management sponsorship of change as critical to the success of organizational change (e.g., Beer, 2021). As previously noted, the most likely explanation for the absence of effect is that the organizational resources have an indirect effect as opposed to a direct effect on change engagement. The non-significant influence of organizational resources on change engagement might also be explained with reference to the impact of proximal versus distal relationships (Albrecht, 2012). Proximal factors influence represent the everyday and routine work experiences of individual employees and teams (Shalley et al., 2000), while distal factors refer to more macro and system-level aspects of the work environment that are more indirectly experienced by individual employee and teams. Consistent with previous research (e.g., Dietz et al., 2004) the results of the present study suggest that employee experiences of change appear to result most strongly from their proximal job level experiences as opposed to organizational level change resources.

Beyond identifying that change-related organizational resources have a strong direct effect on change-related job resources, the study confirmed previous research findings that identified the important role that change-related job resources have on employee attitudes to change. Change involvement, change information, and learning and development opportunities were identified as strong correlates of change engagement. The results therefore corroborate what has long been known regarding factors that influence employee attitudes to change (e.g., Coch and French, 1948).

The study also contributed to the literature by defining and measuring change engagement as a potentially useful change-related construct. Drawing from engagement theory (Bakker and Xanthopoulou, 2013), change engagement was defined as “an enduring and positive work-related psychological state characterized by a genuine enthusiasm and willingness to support, adopt and promote organizational change” (Albrecht et al., 2020, p. 4). As previously noted, the definition reflects the positive energy and active involvement that characterize employee engagement (González-Romá et al., 2006; Macey and Schneider, 2008), and applies them to the context of change. As such, and in contrast to existing constructs such as change readiness, or change commitment (Herscovitch and Meyer, 2002; Bouckenooghe et al., 2009), change engagement is a more high-arousal and high-valence construct. Change-engaged employees will likely be more willing to invest energy and be involved in change, promote organizational change, and strive to engage in change-related behavior.

With respect to the measurement of change engagement, confirmatory factor analyses showed discriminant and convergent validity between the two sub-dimensions of enthusiasm. The correlations between the sub-dimensions did not suggest excessive overlap, and the alpha reliabilities for each sub-dimension clearly exceeded accepted criteria (Nunnally and Bernstein, 1994). Additionally, each dimension was strongly indicated by the higher order construct and the sub-dimensions were differentially predicted by both organizational and job-level change resources in both samples. Table 1 provides researchers and practitioners with change-related measures that have acceptable psychometric properties, and which for the most part generalized across the two samples.

Overall, the results extend current understanding of the role of change-related organizational resources and change-related job resources on employee engagement in change. In part addressing concerns over the lack of theory that underpins organizational change research (By, 2005; Straatmann et al., 2016), the research goes some way toward synthesizing the change and engagement literatures and suggests that “employee engagement in organizational change” is a useful way of understanding organizational change in constantly changing contemporary organizational contexts (Albrecht et al., 2020).

### Practical Implications

The present research suggests practical actions that organizations can take to release the pent-up latent energy that many employees have for constructive organizational change (Beer, 2021). The research suggests that organizations, change leaders, and change agents can usefully focus their attention on the wider organizational system to provide employees with integrated and aligned change-related organizational and job-level resources. When that is the case, employees will more likely be enthusiastic about and involved in change.

In terms of change-related organizational development initiatives, organizations could usefully consider focusing energy and resources on systematically integrating and aligning the five change-related organizational resources and the three change-related job resources included in Figure 2. The results of the present study suggest that interventions to enhance the five change-related organizational resources will likely result in employees feeling positive about their involvement in change, the information they receive about change, and the learning opportunities that present as a consequence of change. Senior leaders, for example, could usefully be coached in how to actively promote, sponsor, resource, and support ongoing organizational change (Albrecht et al., 2020). Consistent with emergent thinking on the leadership of change, such coaching may be directed toward more distributed and collective models of change leadership (e.g., By, 2021). More specifically, senior leaders could be coached on how to develop a supportive climate for change, how to facilitate broad employee involvement and participation in change (Jones et al., 2005), how to consistently and clearly communicate change values and goals (Beer, 2021), and how to model change congruent behaviors, practices and procedures (Kotter, 1990). Such interventions will enable employees to feel more informed, involved, and positive about learning opportunities presented by change. Additionally, HR change supports such as induction training and ongoing skill development could usefully be adopted in order to support employee participation in and support of ongoing organizational change. Similarly, interventions aimed at enhancing system-level change capability, clarity of organizational change goals, and the culture and climate for change will indirectly, through job-level change resources, positively influence employee perceptions of change within their immediate working environment.

More generally, the research provides measures of change-related organizational resources and a measure of employee change engagement that extend the current range of job-level diagnostics and attitudes to change. That is, in addition to assessing the influence of change-related job resources on employee enthusiasm for and involvement in change, survey diagnostics can be administered to assess the extent to which employees perceive the organization has capacity for change, clear organizational change goals, senior leader sponsorship and support for change, HR support, and a climate that is conducive to organizational change. Survey feedback processes can then be used to build on strengths and to identify improvement opportunities to develop organizational change capability.

### Limitations and Future Research Directions

Although the present research has provided new insights into the relationships between organizational resources, change-related job resources, and change engagement, some limitations need to be acknowledged. Firstly, despite the use of quite rigorous confirmatory and structural modeling techniques conducted across two independent samples, cross-sectional data cannot establish causal relations. Future research will usefully involve longitudinal analyses to better understand the causal and reciprocal relationships between the variables modeled.

Secondly, as the data were self-reported, the potential influence of CMV needs to be considered (Podsakoff et al., 2012). However, given that the measurement model was shown to have acceptable fit in both samples, given the small to moderate correlations between the measured constructs, given the modest average reduction in the standardized loadings after the inclusion of a common methods factor, and given the number of statistically significant loadings after the common methods factor was modeled, CMV appears not to be a major concern (Elangovan and Xie, 2000; Podsakoff et al., 2012).

Another limitation centers on the generalizability of the findings. The participants from both samples were working in a range of different organizations. Researchers could usefully sample a range of discrete organizations across different industry sectors to test the generalizability of the measures and the model. Future research could also usefully examine the moderating influence of demographic variables such as age, gender, job tenure, and organizational size on the proposed relationships.

Beyond the suggestions for future research already noted, research could also usefully be directed toward examining the separate, combined, and reciprocal influence of additional resources on change engagement. For example, future research could examine the influence of organizational change culture, psychological safety climate (Dollard and Bakker, 2010), organizational strategic alignment (Biggs et al., 2014), and clarity of organizational values (Denison et al., 2013). Additional change-related job resources such as supervisor and co-worker support for change and change autonomy could also usefully be included in the model. As suggested by Albrecht et al. (2020) and Barrick et al. (2015), researchers could also usefully test how organizational resources influence personal resources such as change self-efficacy, change psychological safety and change meaningfulness. The influence of change-related job demands on change fatigue and change cynicism could also be examined.

## Conclusion

Overall, this study aimed to contribute to the organizational change literature by introducing the construct of employee change engagement, and to test a model showing how change engagement is influenced by change-related organizational and job resources. Although change-related job resources were shown to have the strongest positive association with change engagement, the results support the theory-based model and highlight the role of change-related organizational resources in understanding employee enthusiasm for and involvement in change.

## Data Availability Statement

The raw data supporting the conclusions of this article will be made available by the authors, without undue reservation.

## Ethics Statement

The studies involving human participants were reviewed and approved by the Deakin University Ethics Committee in accord with the Australian Government National Statement on Ethical Conduct in Human Research (2007). The patients/participants provided their written informed consent to participate in this study.

## Author Contributions

SA initiated the conceptualization of the project, collected the data, conducted the analyses, and was responsible for the structure and content of the final manuscript. SC participated in the idea development, data collection, data analyses, and contributed to the structure and content of the final manuscript. ML reviewed the structure and content of the final manuscript and provided support for the publication process. All authors approved the final submitted manuscript.

## Conflict of Interest

SC was employed by the Nous Group. The remaining authors declare that the research was conducted in the absence of any commercial or financial relationships that could be construed as a potential conflict of interest.

## Publisher’s Note

All claims expressed in this article are solely those of the authors and do not necessarily represent those of their affiliated organizations, or those of the publisher, the editors and the reviewers. Any product that may be evaluated in this article, or claim that may be made by its manufacturer, is not guaranteed or endorsed by the publisher.

## Footnotes

1
https://www.prolific.co

## References

[B1] Albrecht S. L. (2012). The influence of job, team and organizational level resources on employee well-being, engagement, commitment and extra-role performance: test of a model. *Int. J. Manpow.* 33 840–853. 10.1108/01437721211268357

[B2] Albrecht S. L. (2021). “Employee engagement and engagement in change: A research agenda,” in *a Research Agenda for Employee Engagement in a Changing World of Work*, eds Meyer J. P. Schneider B. (Cheltenham: Edward Elgar), 155–172.

[B3] Albrecht S. L. Bakker A. B. Gruman J. Macey W. Saks A. (2015). Employee engagement, human resource management practices and competitive advantage. *J. Organ. Eff.* 2 7–35. 10.1108/JOEPP-08-2014-0042

[B4] Albrecht S. L. Breidahl E. Marty A. (2018). Organizational resources, organizational engagement climate, and employee engagement. *Career Dev. Int.* 23 67–85. 10.1108/CDI-04-2017-0064

[B5] Albrecht S. L. Connaughton S. Foster K. Furlong S. Yeow J. (2020). Change engagement, change resources and change demands: a model for positive employee orientations to organizational change. *Front. Psychol.* 11:531944. 10.3389/fpsyg.2020.531944 33240144 PMC7681240

[B6] Allen J. Jimmieson N. L. Bordia P. Irmer B. E. (2007). Uncertainty during organizational change: managing perceptions through communication. *J. Chang. Manag.* 7 187–210. 10.1080/14697010701563379

[B7] Anderson J. C. Gerbing D. W. (1988). Structural equation modeling in practice: a review and recommended two-step approach. *Psychol. Bull.* 103 411–423. 10.1037/0033-2909.103.3.411

[B8] Armenakis A. A. Bedeian A. (1999). Organizational change: a review of theory and research in the 1990s. *J. Manag.* 25 293–315. 10.1177/014920639902500303

[B9] Armenakis A. A. Bernath J. B. Pitts J. P. Walker H. J. (2007). Organizational change recipients’ beliefs scale: development of an assessment instrument. *J. Appl. Behav. Sci.* 43 481–505. 10.1177/0021886307303654

[B10] Armenakis A. A. Harris S. G. Field H. S. (1999). “Making change permanent: A model for institutionalizing change interventions,” in *Research in Organizational Change and Development*, eds Pasmore W. Woodman R. (Greenwich, CT: Jai Press), 97–128.

[B11] Bakker A. B. Demerouti E. (2014). “Job demands-resources theory,” in *Wellbeing: A Complete Reference Guide*, eds Chen P. Y. Cooper C. L. (Hoboken: Wiley-Blackwell), 37–64.

[B12] Bakker A. B. Demerouti E. (2017). Job demands-resources theory: taking stock and looking forward. *J. Occup. Health Psychol.* 22 273–285. 10.1037/ocp0000056 27732008

[B13] Bakker A. B. Xanthopoulou D. (2013). Creativity and charisma among female leaders: the role of resources and work engagement. *Int. J. Hum. Resour.* 24 2760–2779. 10.1080/09585192.2012.751438

[B14] Barrick M. R. Thurgood G. R. Smith T. A. Courtright S. H. (2015). Collective organizational engagement: linking motivational antecedents, strategic implementation, and firm performance. *Acad. Manag. J.* 58 111–135. 10.5465/amj.2013.0227

[B15] Bartunek J. M. Rousseau D. M. Rudolph J. W. DePalma J. A. (2006). On the receiving end: sensemaking, emotion, and assessments of an organizational change initiated by others. *J. Appl. Behav. Sci.* 42 182–206. 10.1177/0021886305285455

[B16] Beer M. (1999). “Leading learning and learning to lead: An action learning approach to developing organizational fitness,” in *The leader’s change handbook: An essential guide to setting direction and taking action*, eds Conger J. A. Spreitzer G. M. Lawler E. E. III (Hoboken, NJ: Jossey-Bass), 127–161.

[B17] Beer M. (2021). Reflections: towards a normative and actionable theory of planned organizational change and development. *J. Chang. Manag.* 21 14–29. 10.1080/14697017.2021.1861699

[B18] Beer M. Nohria N. (2000). Cracking the code of change. *Harv. Bus. Rev.* 78 133–141. 11183975

[B19] Beus J. M. Solomon S. J. Taylor E. C. Esken C. A. (2020). Making sense of climate: a meta-analytic extension of the competing values framework. *Organ. Psychol. Rev.* 10 136–168. 10.1177/2041386620914707

[B20] Biggs A. Brough P. Barbour J. P. (2014). Strategic alignment with organizational priorities and work engagement: a multi-wave analysis. *J. Organ. Behav.* 35 301–317. 10.1002/job.1866

[B21] Bordia P. Hobman E. Jones E. Gallois C. Callan V. J. (2004). Uncertainty during organizational change: types, consequences, and management strategies. *J. Bus. Psychol.* 18 507–532. 10.1023/B:JOBU.0000028449.99127.f7

[B22] Bouckenooghe D. Devos G. Van den Broeck H. (2009). Organizational change questionnaire-climate of change, processes, and readiness: development of a new instrument. *J. Psychol.* 143 559–599. 10.1080/00223980903218216 19957876

[B23] Browne M. W. Cudeck R. (1993). “Alternative ways of assessing model fit,” in *Testing Structural Equation Models*, eds Bollen K. A. Long J. S. (Thousand Oaks: Sage), 136–162.

[B24] Burke W. W. (2011). A perspective on the field of organization development and change: The Zeigarnik Effect. *J. Appl. Behav. Sci.* 47 143–167. 10.1177/0021886310388161

[B25] Burke W. W. (2014). “Organizational change,” in *The Handbook of Organizational Climate and Culture: Antecedents, Consequences, and Practice*, eds Schneider B. Barbera K. (Oxford: Oxford University Press), 457–483.

[B26] Burnes B. (2004). *Managing Change: A Strategic Approach to Organizational Dynamics* (4th Ed.). Hoboken: Prentice Hall.

[B27] Burnes B. (2011). Introduction: why does change fail, and what can we do about it? *J. Chang. Manag.* 11 445–450. 10.1080/14697017.2011.630507

[B28] By R. T. (2005). Organizational change management: a critical review. *J. Chang. Manag.* 5 369–380. 10.1080/14697010500359250

[B29] By R. T. (2021). Leadership: in pursuit of purpose. *J. Chang. Manag.* 21 30–44. 10.1080/14697017.2021.1861698

[B30] Byrne B. M. (2001). Structural equation modeling: perspectives on the present and the future. *Int. J. Test.* 1 327–334. 10.1080/15305058.2001.9669479

[B31] Choi M. (2011). Employees’ attitudes toward organizational change: a literature review. *Hum. Resour. Manag.* 50 4679–4500. 10.1002/hrm.20434

[B32] Coch L. French J. R. P. (1948). Overcoming resistance to change. *Hum. Relat.* 1 512–532. 10.1177/001872674800100408

[B33] Costanza D. P. Blacksmith N. Coats M. R. Severt J. B. DeCostanza A. H. (2016). The effect of adaptive organizational culture on long-term survival. *J. Bus. Psychol.* 31 361–381. 10.1007/s10869-015-9420-y

[B34] Crawford E. R. LePine J. Rich B. (2010). Linking job demands and resources to employee engagement and burnout: a theoretical extension and meta-analytic test. *J. Appl. Psychol.* 95 834–848. 10.1037/a0019364 20836586

[B35] de Jonge J. Dormann C. (2006). Stressors, resources, and strains at work: a longitudinal test of the triple-match principle. *J. Appl. Psychol.* 91 1359–1374. 10.1037/0021-9010.91.5.1359 17100490

[B36] Denison D. R. Ko I. Kotbra L. Nieminen L. (2013). Drive an innovative culture. *Chief Learn. Off.* 70–72.

[B37] Devos G. Buelens M. Bouckenooghe D. (2007). Contribution of content, context, and process to understanding openness to organizational change: two experimental simulation studies. *J. Soc. Psychol.* 147 607–630. 10.3200/SOCP.147.6.607-630 18314790

[B38] Dietz J. Pugh S. D. Wiley J. W. (2004). Service climate effects on customer attitudes: an examination of boundary conditions. *Acad. Manag. J.* 47 81–92. 10.2307/20159561

[B39] Dollard M. F. Bakker A. B. (2010). Psychosocial safety climate as a precursor to conducive work environments, psychological health problems, and employee engagement. *J. Occup. Organ. Psychol.* 83 579–599. 10.1348/096317909X470690

[B40] Eby L. T. Adams D. M. Russell J. E. A. Gaby S. H. (2000). Perceptions of organizational readiness for change: factors related to employees’ reactions to the implementation of team-based selling. *Hum. Relat.* 53 419–442. 10.1177/0018726700533006

[B41] Eisenhardt K. M. Martin J. A. (2000). Dynamic capabilities: what are they? *Strateg. Manag. J.* 21 961–979. 10.1002/1097-0266

[B42] Elangovan A. R. Xie J. L. (2000). Effects of perceived power of supervisor on subordinate work attitudes. *Leadersh. Dev. J.* 21 319–328. 10.1108/01437730010343095

[B43] Elving W. J. L. (2005). The role of communication in organizational change. *Corp. Commun.* 10 129–138. 10.1108/13563280510596943

[B44] Feldman M. S. (2000). Organizational routines as a source of continuous change. *Organ. Sci.* 11 611–629. 10.1287/orsc.11.6.611.12529 19642375

[B45] Ford J. Ford L. Polin B. (2021). Leadership in the implementation of change: functions, sources, and requisite variety. *J. Chang. Manag.* 21 87–119. 10.1080/14697017.2021.1861697

[B46] Frijda N. H. (1986). *The Emotions.* Cambridge: Cambridge University Press.

[B47] Fugate M. (2012). The impact of leadership, management, and HRM on employee reactions to organizational change. *Res. Pers. Hum. Resour. Manag.* 31 177–208. 10.1108/S0742-730120120000031007

[B48] Fugate M. Prussia P. E. Kinicki A. J. (2012). Managing employee withdrawal during organizational change: the role of threat appraisal. *J. Manag.* 38 890–914. 10.1177/0149206309352881

[B49] Goh Z. Eva N. Kiazad K. Jack G. A. De Cieri H. Spreitzer G. M. (2022). An integrative multilevel review of thriving at work: assessing progress and promise. *J. Organ. Behav.* 43, 197–213. 10.1002/job.2571

[B50] González-Romá V. Schaufeli W. Bakker A. Lloret S. (2006). Burnout and work engagement: independent factors or opposite poles? *J. Vocat. Behav.* 68 165–174. 10.1016/j.jvb.2005.01.003

[B51] Gould-Williams J. Davies F. (2005). Using social exchange theory to predict the effects of HRM practice on employee outcomes. *Public Manag. Rev.* 7 1–24. 10.1080/1471903042000339392

[B52] Halbesleben J. R. B. (2010). “A meta-analysis of work engagement: relationships with burnout, demands, resources and consequences,” in *Work Engagement: A Handbook of Essential Theory and Research*, eds Bakker A. Leiter M. (Hove: Psychology Press), 102–117.

[B53] Heene M. Hilbert S. Draxler C. Ziegler M. Bühner M. (2011). Masking misfit in confirmatory factor analysis by increasing unique variances: a cautionary note on the usefulness of cutoff values of fit indices. *Psychol. Methods* 16 319–336. 10.1037/a0024917 21843002

[B54] Herscovitch L. Meyer J. P. (2002). Commitment to organizational change: extension of a three-component model. *J. Appl. Psychol.* 87 474–487. 10.1037/0021-9010.87.3.474 12090605

[B55] Hobfoll S. E. Halbesleben J. Neveu J.-P. Westman M. (2018). Conservation of resources in the organizational context: the reality of resources and their consequences. *Annu. Rev. Organ. Psychol. Organ. Behav.* 5 103–130. 10.1146/annurev-orgpsych-032117-104640

[B56] Hu L. Bentler P. M. (1998). Fit indices in covariance structure modeling: sensitivity to under-parameterized model misspecification. *Psychol. Methods* 3, 424–453. 10.1037/1082-989X.3.4.424

[B57] Hu L. Bentler P. M. (1999). Cutoff criteria for fit indexes in covariance structure analysis: conventional criteria versus new alternatives. *Struct. Equ. Model.* 6 1–55. 10.1080/10705519909540118

[B58] Hughes M. (2011). Do 70 per cent of all organizational change initiatives really fail? *J. Chang. Manag.* 11 451–464. 10.1080/14697017.2011.630506

[B59] Jackson D. L. Gillaspy J. A. Jr. Purc-Stephenson R. (2009). Reporting practices in confirmatory factor analysis: an overview and some recommendations. *Psychol. Methods* 14 6–23. 10.1037/a0014694 19271845

[B60] Jones R. A. Jimmieson N. L. Griffiths A. (2005). The impact of organizational culture and reshaping capabilities on change implementation success: the mediating role of readiness for change. *J. Manag. Stud.* 42 361–386. 10.1111/j.1467-6486.2005.00500.x

[B61] Jöreskog K. G. Sörbom D. (1993). *Lisrel 8: Structural Equations Modeling with SIMPLIS Command Language.* Mahwah: Lawrence Erlbaum Associates, Inc.

[B62] Judge W. Douglas T. (2009). Organizational change capacity: the systematic development of a scale. *J. Organ. Change Manag.* 22 635–649. 10.1108/09534810910997041

[B63] Kahn W. A. (1990). Psychological conditions of personal engagement and disengagement at work. *Acad. Manag. J.* 33 692–724. 10.5465/256287

[B64] Karasvirta S. Teerikangas S. (2022). Change organizations in planned change – a closer look. *J. Chang. Manag.* 22 163–201. 10.1080/14697017.2021.2018722

[B65] Kotter J. P. (1990). *A Force for Change: How Leadership Differs from Management.* Free Press House: Free Press.

[B66] MacCallum R. C. Browne M. W. Sugawara H. M. (1996). Power analysis and determination of sample size for covariance structure modelling. *Psychol. Methods* 1 130–149. 10.1037/1082-989X.1.2.130

[B67] Macey W. H. Schneider B. (2008). The meaning of employee engagement. *Ind. Organ. Psychol.* 1 3–30. 10.1111/j.1754-9434.2007.0002.x

[B68] Mathews B. Linski C. M. (2016). Shifting the paradigm: reevaluating resistance to organizational change. *J. Organ. Chang. Manag.* 29 963–972. 10.1108/JOCM-03-2016-0058

[B69] Meyer C. B. Stensaker I. G. (2006). Developing capacity for change. *J. Chang. Manag.* 6 217–231. 10.1080/14697010600693731

[B70] Miller D. (2004). Building sustainable change capability. *Ind. Commer. Train.* 36 9–12. 10.1108/00197850410516058

[B71] Miller V. D. Johnson J. R. Grau J. (1994). Antecedents to willingness to participate in a planned organizational change. *J. Appl. Commun. Res.* 22 59–80. 10.1080/00909889409365387

[B72] Milliken F. J. (1987). Three types of perceived uncertainty about the environment: state, effect, and response uncertainty. *Acad. Manag. Rev.* 12 133–143. 10.5465/amr.1987.4306502

[B73] Nunnally J. C. Bernstein I. H. (1994). *Psychometric Theory.* New York: McGraw-Hill.

[B74] Oreg S. (2006). Personality, context, and resistance to organizational change. *Eur. J. Work Organ. Psychol.* 15 73–101. 10.1080/13594320500451247

[B75] Oreg S. Bartunek J. Lee G. Do B. (2018). An affect-based model of recipients’ responses to organizational change events. *Acad. Manag. Rev.* 43 65–86. 10.5465/amr.2014.0335

[B76] Oreg S. Vakola M. Armenakis A. (2011). Change recipients’ reactions to organizational change: a 60-year review of quantitative studies. *J. Appl. Behav. Sci.* 47 461–524. 10.1177/0021886310396550

[B77] Paré G. Sicotte C. Poba-Nzaou P. Balouzakis G. (2011). Clinicians’ perceptions of organizational readiness for change in the context of clinical information system projects: insights from two cross-sectional surveys. *Implement. Sci.* 6:15. 10.1186/1748-5908-6-15 21356080 PMC3056827

[B78] Pasmore W. A. (2011). “Tipping the balance: Overcoming persistent problems in organizational change,” in *Research in Organizational Change and Development*, eds Shani A. Woodman R. Pasmore W. (Greenwich, CT: Jai Press), 259–292.

[B79] Patterson M. G. West M. A. Shackleton V. J. Dawson J. F. Lawthom R. Maitlis S. (2005). Validating the organizational climate measure: links to managerial practices, productivity and innovation. *J. Organ. Behav.* 26 379–408. 10.1002/job.312

[B80] Pearce C. L. Sims H. P. (2002). Vertical versus shared leadership as predictors of the effectiveness of change management teams: an examination of aversive, directive, transactional, transformational, and empowering leader behaviors. *Group Dyn. Theory Res. Pract.* 171 172–197. 10.1037/1089-2699.6.2.172

[B81] Podsakoff P. M. MacKenzie S. B. Podsakoff N. P. (2012). Sources of method bias in social science research and recommendations on how to control it. *Ann. Rev. Psychol.* 63 539–569. 10.1146/annurev-psych-120710-100452 21838546

[B82] Putnick D. L. Bornstein M. H. (2016). Measurement invariance conventions and reporting: the state of the art and future directions for psychological research. *Dev. Rev.* 41 71–90. 10.1016/j.dr.2016.06.004 27942093 PMC5145197

[B83] Rafferty A. E. Jimmieson N. L. Armenakis A. A. (2013). Change readiness: a multilevel review. *J. Manag.* 39 110–135. 10.1177/0149206312457417

[B84] Rafferty A. E. Minbashian A. (2019). Cognitive beliefs and positive emotions about change: relationships with employee change readiness and change supportive behaviors. *Hum. Relat.* 72 1623–1650. 10.1177/0018726718809154

[B85] Rafferty A. E. Simons R. H. (2005). An examination of the antecedents of readiness for fine-tuning and corporate transformation changes. *J. Bus. Psychol.* 20 325–350. 10.1007/s10869-005-9013-2

[B86] Rich B. L. LePine J. A. Crawford E. R. (2010). Job engagement: antecedents and effects on job performance. *Acad. Manag. J.* 53 617–635. 10.5465/AMJ.2010.51468988

[B87] Russell J. A. (2003). Core affect and the psychological construction of emotion. *Psychol. Rev.* 110 145–172. 10.1037/0033-295X.110.1.145 12529060

[B88] Sagiv L. Schwartz S. H. (2007). Cultural values in organisations: insights for Europe. *Eur. J. Int. Manag.* 1 176–190. 10.1504/EJIM.2007.014692 35009967

[B89] Schneider B. Brief A. Guzzo R. (1996). Creating a climate and culture for sustainable organizational change. *Organ. Dyn.* 24 7–19. 10.1016/S0090-2616(96)90010-8

[B90] Schweiger D. M. Denisi A. S. (1991). Communication with employees following a merger: a longitudinal field experiment. *Acad. Manag. J.* 34 110–135. 10.5465/256304

[B91] Shalley C. E. Gilson L. L. Blum T. C. (2000). Matching creativity requirements and the work environment: effects on satisfaction and intent to turnover. *Acad. Manag. J.* 43 215–224. 10.5465/1556378

[B92] Shea C. M. Jacobs S. R. Esserman D. A. Bruce K. Weiner B. J. (2014). Organizational readiness for implementing change: a psychometric assessment of a new measure. *Implement. Sci.* 9:7. 10.1186/1748-5908-9-7 24410955 PMC3904699

[B93] Straatmann T. Kohnke O. Hattrup K. Mueller K. (2016). Assessing employees’ reactions to organizational change: an integrative framework of change-specific psychological factors. *J. Appl. Behav. Sci.* 52 265–295. 10.1177/0021886316655871

[B94] Ten Have S. Ten Have W. Huijsmans A.-B. Otto M. (2017). “Introduction,” in *Reconsidering Change Management: Applying Evidence-Based Insights in Change Management Practice*, eds Ten Have S. Ten Have W. Huijsmans A.-B. Otto M. (Milton Park: Routledge), 1–14.

[B95] Thundiyil T. G. Chiaburu D. S. Oh I.-S. Banks G. C. Peng A. C. (2015). Cynical about change? A preliminary meta-analysis and future research agenda. *J. Appl. Behav. Sci.* 51 429–450. 10.1177/0021886315603122

[B96] Tonidandel S. LeBreton J. M. (2015). RWA Web: a free, comprehensive, web-based, and user-friendly tool for relative weight analyses. *J. Bus. Psychol.* 30 207–216. 10.1007/s10869-014-9351-z

[B97] Tonidandel S. LeBreton J. M. Johnson J. W. (2009). Determining the statistical significance of relative weights. *Psychol. Methods* 14 387–399. 10.1037/a0017735 19968399

[B98] Tsaousis I. Vakola M. (2018). “Measuring change recipients’ reactions: The development and psychometric evaluation of the CRRE scale,” in *Organizational Change: Psychological Effects and Strategies for Coping*, eds Vakola M. Petrou P. (Milton Park: Routledge), 114–127.

[B99] Van Emmerik I. J. H. Bakker A. B. Euwema M. C. (2009). Explaining employees’ evaluations of organizational change with the job-demands resources model. *Career Dev. Int.* 14 594–613. 10.1108/13620430910997312

[B100] Vandenberg R. J. Lance C. E. (2000). A review and synthesis of the measurement invariance literature: suggestions, practices, and recommendations for organizational research. *Organ. Res. Methods* 2 4–69. 10.1177/109442810031002

[B101] Walter S. L. Seibert S. E. Goering D. O’Boyle E. H. (2019). A tale of two sample sources: do results from online panel data and conventional data converge? *J. Bus. Psychol.* 34 425–452. 10.1007/s10869-018-9552-y

[B102] Wanberg C. R. Banas J. T. (2000). Predictors and outcomes of openness to changes in a reorganizing workplace. *J. Appl. Psychol.* 85 132–142. 10.1037/0021-9010.85.1.132 10740964

[B103] Wanous J. P. Reichers A. E. Austin J. T. (2000). Cynicism about organizational change: measurement, antecedents, and correlates. *Group Organ. Manag.* 25 132–153. 10.1177/1059601100252003

[B104] Weick K. E. (2000). “Emergent change as a universal in organizations,” in *Breaking the Code of Change*, eds Beer M. Nohria N. (Boston: Harvard Business School Press), 223–242.

[B105] Weiner B. J. (2009). A theory of organizational readiness for change. *Implement. Sci.* 4:67. 10.1186/1748-5908-4-67 19840381 PMC2770024

[B106] Weiner B. J. Amick H. Less S. Y. (2008). Conceptualization and measurement of organizational readiness for change: a review of the literature in health services research and other fields. *Med. Care Res. Rev.* 65 379–436. 10.1177/1077558708317802 18511812

[B107] Whelan-Berry K. S. Somerville K. A. (2010). Linking change drivers and the organizational change process: a review and synthesis. *J. Chang. Manag.* 10 175–193. 10.1080/14697011003795651

[B108] Yukl G. Mahsud R. Prussia G. Hassan S. (2019). Effectiveness of broad and specific leadership behaviors. *Pers. Rev.* 48 774–783. 10.1108/PR-03-2018-0100

